# Somatosensation Evoked by Cortical Surface Stimulation of the Human Primary Somatosensory Cortex

**DOI:** 10.3389/fnins.2019.01019

**Published:** 2019-09-24

**Authors:** St. Clair Kirin, Takufumi Yanagisawa, Satoru Oshino, Kohtaroh Edakawa, Masataka Tanaka, Haruhiko Kishima, Yukio Nishimura

**Affiliations:** ^1^Department of Developmental Physiology, National Institute for Physiological Sciences, Okazaki, Japan; ^2^Department of Physiological Sciences, School of Life Sciences, The Graduate University for Advanced Studies (SOKENDAI), Hayama, Japan; ^3^Department of Neurosurgery, Graduate School of Medicine Osaka University, Suita, Japan; ^4^Center for Information and Neural Networks, National Institute of Information and Communications Technology, Suita, Japan; ^5^Institute for Advanced Co-Creation Studies, Osaka University, Suita, Japan; ^6^Neural Prosthesis Project, Department of Dementia and Higher Brain Function, Tokyo Metropolitan Institute of Medical Science, Tokyo, Japan

**Keywords:** artificial sensation, primary somatosensory cortex, electrocorticography, electrical stimulation, human

## Abstract

Electrical stimulation of the primary somatosensory cortex using intracranial electrodes is crucial for the evocation of artificial somatosensations, typically tactile sensations associated with specific regions of the body, in brain-machine interface (BMI) applications. The qualitative characteristics of these artificially evoked somatosensations has been well documented. As of yet, however, the quantitative aspects of these evoked somatosensations, that is to say the quantitative relationship between intensity of electrical stimulation and perceived intensity of the resultant somatosensation remains obscure. This study aimed to explore this quantitative relationship by surface electrical stimulation of the primary somatosensory cortex in two human participants undergoing electrocorticographic monitoring prior to surgical treatment of intractable epilepsy. Electrocorticogram electrodes on the primary somatosensory cortical surface were stimulated with varying current intensities, and a visual analogue scale was employed to provide a quantitative measure of intensity of the evoked sensations. Evoked sensations included those of the thumb, tongue, and hand. A clear linear relationship between current intensity and perceived intensity of sensation was observed. These findings provide novel insight into the quantitative nature of primary somatosensory cortex electrical stimulation-evoked sensation for development of somatosensory neuroprosthetics for clinical use.

## Introduction

Artificial somatosensory feedback will be critical for execution of fine motor control using brain-machine interface (BMI). Under natural conditions, the brain relies on online somatosensory feedback to guide limb movements. Those with impaired somatosensory function often make gross errors in motor output (Gandevia et al., 1990; Sainburg et al., 1993, 1995; Darian-Smith and Ciferri, 2005). Moreover, spinal cord injury and stroke, which are major targets for clinical BMI (Yanagisawa et al., 2011, 2012), are common causes of somatosensory as well as motor impairment (Yanagisawa et al., 2016). Artificial somatosensory feedback via neuroprosthesis is necessary in order to accomplish natural movements with a clinically plausible BMI (Suminski et al., 2010; Dadarlat et al., 2015; Pistohl et al., 2015; Schiefer et al., 2016).

Human and animal studies have indicated that somatosensory function can be restored using neuroprosthetics. Electrical stimulation of S1 evokes artificial somatosensation (Penfield and Boldrey, 1937; Lueders et al., 1983; Johnson et al., 2013). Some studies using monkeys implanted with intra-cortical micro-electrodes demonstrated that electrical stimulation through the electrodes allowed the monkey to discriminate different stimulation frequencies to S1 (Romo et al., 1998; O’Doherty et al., 2011). Further work in humans using electrodes implanted in the somatosensory cortex allowed patients with lost sensation in the hand to discriminate sensations on different fingers (Johnson et al., 2013; Flesher et al., 2016; Hiremath et al., 2017; Caldwell et al., 2019). For both intracortical microelectrodes and surface planar electrodes, electrical stimulation has been successfully used to evoke sensation of the upper limbs dependent on stimulus parameters such as frequency and intensity (Flesher et al., 2016; Hiremath et al., 2017). However, the nature of this artificially evoked somatosensation for different body parts, such as the hand and tongue, and its relationship to the physiological somatosensation experienced as a result of peripheral sensory inputs have yet to be fully elucidated.

In order to design somatosensory neuroprosthesis for long term use in human patients, it is critical to fully characterize the subjective sensation evoked by S1 stimulation. However, a quantitative psychophysical analysis of these S1 electrical stimulation-evoked sensations for different body parts remain obscure. The present study employed a visual analogue scale (VAS) (Zealley and Aitken, 1969; McCormack et al., 1988; Bijur et al., 2001) to quantify the strength of somatosensation experienced by two participants, who were undergoing ECoG monitoring prior to surgical treatment of intractable epilepsy, during electrical stimulation of S1, in order to elucidate the quantitative relationship between S1 stimulation current intensity and perceived intensity of evoked sensation. It was found that stimulation current intensity shares a linear relationship with perceived intensity of somatosensation, within the ranges of current intensities tested, for both the hand and tongue. These results advance toward the goal of understanding the subjective experience of S1 electrical stimulation in order to realize a clinically plausible BMI.

## Materials and Methods

### Participants

Two participants undergoing pre-operative electrocorticographic (ECoG) examination for surgical treatment of intractable epilepsy took part in this study. This experimental protocol was approved by the Ethics Committees of the Osaka University Hospital (Approval No. 14353), the National Institutes for Physiological Sciences (Approval No. 16B004), and the Tokyo Metropolitan Institute of Medical Science (Approval No. 17-2) and carried out in accordance with the Declaration of Helsinki. Participants or their guardians provided written, informed consent to the experimental procedures and to the use of their data for academic study.

**FIGURE 1:**
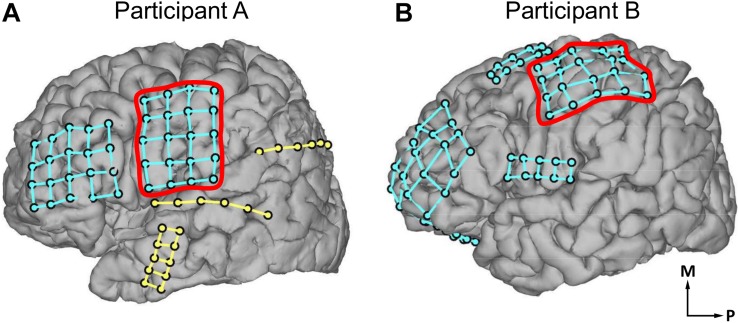
Locations of electrocorticogram electrodes on participants’ cortical surfaces as determined by coregistration of MRI and CT images. Electrode locations are visualized with dots, with different electrode grids distinguished by the presence of grid lines between those dots. **(A)** Electrode locations in participant A. **(B)** Electrode locations in participant B. In **(A,B)**, red outlines indicate the electrode grids analyzed in the current study. The outlined electrode grid was on the left hemisphere of the brain for participant A, and the right hemisphere for participant B; participant B’s data has been mirrored throughout this report for comparison. Axes labels indicate medial (M) and posterior (P) directions.

Participant A, age 35–40 years old, was undergoing treatment for epilepsy due to cavernous malformation on his left precentral gyrus. Participant B, age 15–20 years old, was undergoing treatment for right frontal lobe epilepsy. Subdural ECoG arrays were implanted across the primary somatosensory (S1) and primary motor (M1) cortices. Participant A was implanted with ECoG arrays on the left hemisphere (Figure 1A) and participant B (Figure 1B) on the right hemisphere. Throughout this report, participant B’s data is mirrored to match participant A’s for the sake of comparison. The ECoG electrodes used in this study were implanted exclusively under clinical considerations for the treatment of participants’ diseases. Electrical stimulation to the sensorimotor cortex was performed under the clinical necessity to map the sensorimotor cortex.

### Electrode Localization

**FIGURE 2:**
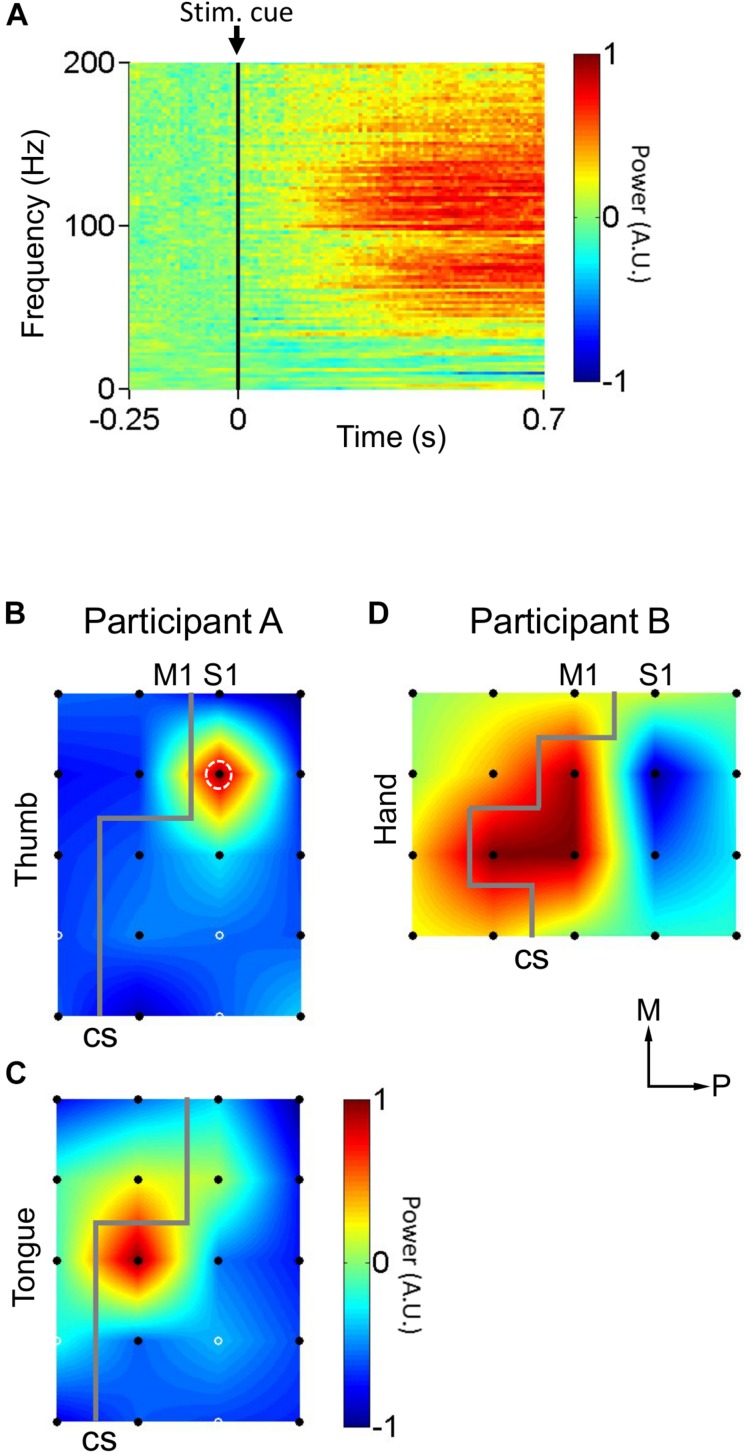
Cortical responses to peripheral mechanical stimulation. **(A)** Average cortical responses upon mechanical stimulation of the thumb in participant A (*n* = 50 trials). Participant A’s thumb were rubbed by a cotton swab. Black vertical lines indicate stimulus instruction onset (Stim. cue). **(B)** Distribution of cortical responses (*n* = 50 trials) to mechanical stimulation of the thumb in participant A. White dashed circle indicates the electrode whose cortical response is detailed in **(A)**. **(C)** Distribution of cortical responses (*n* = 50 trials) to mechanical stimulation of the tongue in participant A. **(D)** Distribution of cortical responses (*n* = 40 trials) to mechanical stimulation of the hand in participant B. Gray lines indicate estimated location of central sulci (CS). Black filled circles indicate electrodes where cortical responses were calculated. White solid circles indicate electrodes where the signal was corrupted by noise, and for where for display purposes the cortical response was taken as the mean of the neighboring electrodes’ responses. Axes labels indicate medial (M) and posterior (P) directions. The color scale in **(B–D)** is the same as in **(A)**. A.U. stands for arbitrary units.

**FIGURE 3:**
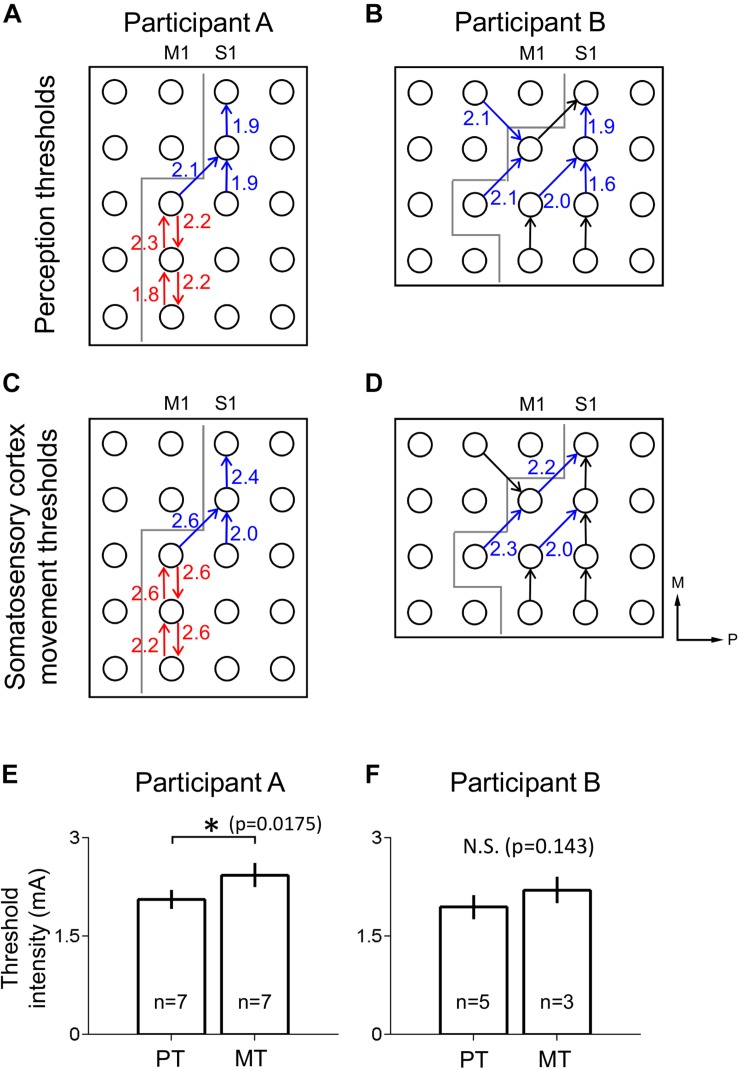
Thresholds for perception and movement evoked by S1 stimulation. **(A,B)** Perception thresholds (PT) at different electrodes for participants A **(A)** and B **(B)** in mA. Arrows point from anode to cathode indicating stimulation polarity (blue: the electrode pairs which induced thumb sensation, red: the electrode pairs which induced tongue sensation, black: no response). Gray line indicates estimated location of central sulcus, dividing M1 and S1. **(C,D)** As in **(A,B)**, but with movement threshold (MT) under S1 stimulation. Axes labels indicate medial (M) and posterior (P) directions for **(A–D)**. **(E)** Comparison of perception (PT) and movement (MT) thresholds in S1 for participant A using a two-tailed Mann–Whitney *U*-test with a significance criterion of *p* < 0.05. Bars and error bars indicate mean and standard error respectively. **(F)** As in **(E),** for participant B. ^∗^Indicates significance at the 0.05 level, and N.S. indicates non-significance.

The ECoG electrode arrays analyzed in this study were composed of grids of 20 planar-surface 3 mm diameter platinum electrodes with 1 cm grid spacing. S1 and M1 mapping and identification of electrode location were performed based on preoperative MRI scans, postoperative CT scans, and neurophysiological evidence. First, the preoperative MRI scans were coregistered with postoperative CT scans using established techniques (Dykstra et al., 2012) via EpiSurg software in an anatomical assessment of electrode location (Figure 1). To identify the central sulcus and to map and identify the locations of electrodes on S1 and M1 (Figures 2B–D, 3A–D, 5A,B), peripheral transcutaneous electrical stimulation and/or mechanical stimulation to the surface of the body was/were applied. Peripheral mechanical stimulation experiments were carried out for both participants while recording ECoG signals. These signals were analyzed to find electrodes that detected an increase in neural activity a response to the peripheral mechanical stimulation, and thus to help ascertain the location of the electrodes with S1 (Figure 2). Participant A also underwent transcutaneous electrical stimulation of the median nerve, which innervates the palmar surface of the thumb, as an additional localization technique. By analyzing the results of obtained by these various experiments together, the locations of the electrodes relative to the central sulcus, S1, and M1 were ascertained. These peripheral mechanical stimulation experiments are explained in more detail below.

### Peripheral Mechanical Stimulation

To identify S1 somatotopy, peripheral mechanical stimulation tests were performed while recording participants’ cortical responses. Every 2 s, the software Presentation^®^ (Neurobehavioral Systems, Albany, CA, United States) generated a TTL pulse signaling stimulus instruction onset (Stim. Cue in Figure 2A) and presented the experimenter with one of two instructions: “Stim” or “Rest.” These indicated whether to mechanically stimulate the participant or to refrain from doing so, respectively. “Stim” and “Rest” instructions were presented randomly. During “Stim” trials, Participant A’s thumb (*n* = 50 trials) or tongue (*n* = 50 trials) were rubbed by a cotton swab, in two separate tests. Participant B’s hand was tapped by the fingers of an experimenter (*n* = 40). The TTL pulses marking stimulus instruction onset (stim. Cue in Figure 2A), the point at which the experimenter was presented with the “Stim” or “Rest” instruction, were recorded alongside the physiological data described below and used to align said physiological data indirectly with the onset of the peripheral mechanical stimulation for each 2 s trial.

During the peripheral mechanical stimulation test, ECoG signals were recorded at 1000 Hz via EEG-2000 (Nihon Kohden, Tokyo, Japan). The ECoG signals during the mechanical stimulation were analyzed from -250 ms before to 750 ms after each stimulus instruction onset. The signals for each trial were transformed into the frequency-time domain through a 512 point (512 ms at a 1000 Hz sampling rate) fast Fourier transform (FFT) sliding window that passed down the length of each trial. For speed of computation, FFT was only calculated for every 10th point of the data. This resulted in a 2-dimensional time series representing neural activity, represented in logarithmic scale, over time across the range of frequencies between 0 and 200 Hz. For each frequency in this range, the baseline activity, defined as the mean value of that particular frequency’s activity during the baseline time window of -250 to 0 ms prior to stimulus instruction onset, was calculated. This frequency-specific baseline value was then subtracted from the corresponding frequency-time signal, for every individual frequency, in order to normalize the data relative to the pre-stimulus baseline activity (Figure 2A). Then, the neural response to the mechanical stimulation was calculated as the average neural activity between 200 and 700 ms post stimulus instruction onset and between 80 and 160 Hz; the high-gamma activity band associated with activation in response to somatosensory stimulation in S1 (Figures 2B–D). Power was normalized within each experiment to the range −1 to 1, in arbitrary units, such that the electrode with the highest neural response to mechanical stimulation was 1 and the lowest was −1, for purposes of simplicity in the localization process.

### S1 Electrical Stimulation

Pairs of electrodes in which at least one electrode was located on S1 were selected based on the results of the anatomical electrode localization. These electrode pairs were electrically stimulated one at a time at a range of current intensities with bipolar 50 Hz pulse trains of 200 μs biphasic pulse width pulses lasting for 3 s each. During the stimulation test, the ECoG signals of all non-stimulated electrodes were monitored to assess the presence of after-discharges. If after-discharges occurred following stimulation, the examination was stopped until a few minutes after the after-discharges disappeared, in order to prevent evoking an epileptic seizure. In general, stimulus current intensity was incrementally increased from low to high. Current intensity ranged from 1.0 to 3.2 mA in participant A and from 0.5 to 3.5 mA in participant B. Notably, we did not perform catch trials (stimulation with 0 mA), although the sensory threshold might be biased without the catch trials. Because we did this experiment as a part of the clinical evaluation, making the implementation of the catch trials difficult. The initial maximum current intensity was set at 3.0 mA for both participants, but when after-discharges were not observed, current intensity was increased beyond this limit after getting permission from the doctors caring for the participants. The current intensity was set to less than 10.0 mA, which corresponds to the charge of 2 μC/phase and charge density of 28.3 μC/phase^∗^cm^2^, so as not to exceed the Shannon criteria (1.75 < 1.85; Shannon, 1992). A reversal of polarity in an electrode pair was considered a distinct electrode pair; any two electrodes could produce two electrode pairs taking polarity into account. Any movement apparently evoked by the stimulation was recorded via video. Furthermore, experimenters visually observing the participant during the electrical stimulation watched for any movements apparently evoked by the stimulation and made note of the qualitative nature of said movements.

During this test, participants were instructed to verbally describe the sensations they felt upon stimulation and to mark on a 100 mm VAS the intensity of any sensations they felt upon stimulation. The VAS was a horizontal line with the left side endpoint labeled “no sensation” and the right endpoint labeled “strongest sensation”. Participant A was instructed to mark the weakest perceivable sensation at a pre-marked point 10 mm from the left “no sensation” endpoint. This was not done with participant B.

The lowest stimulus current to a given electrode pair at which participants gave a VAS score above 0 mm was defined as the perception threshold (PT) for that electrode pair. The lowest stimulus current to a given electrode pair that apparently evoked visible movement in the participant was defined as the movement threshold (MT) for that electrode pair.

### Analysis of VAS Response Characteristics

VAS scores corresponding to S1 electrical stimulation-evoked sensation were linearly fitted for each electrode pair. Electrode pairs that lacked sub-threshold data, defined as current intensities that resulted in 0 mm VAS scores, or that lacked any VAS score above 0 mm, indicating no sensation was perceived, were ignored for this analysis. For fitting purposes, only the highest sub-threshold current intensity was used to calculate the best fit line. The slopes of these best fit lines were defined as the sensitivities of VAS score to current intensity. Electrode pairs in participant A were divided into thumb-related and tongue-related electrode pairs based on verbal descriptions of the electrically evoked sensations given by that participant.

### Statistics

PT and MT were tested for mean difference, in participant A (PT: *n* = 7 electrode pairs, MT: *n* = 7), participant B (PT: *n* = 5, MT: *n* = 3), and in the population of both participants (PT: *n* = 12, MT: *n* = 10), using two-tailed Mann–Whitney *U*-tests with a significance criterion of *p* < 0.05. This non-parametric test was selected due to its applicability to data with small sample sizes.

Sensitivities of thumb-related (*n* = 3) and tongue-related (*n* = 4) electrode pairs in participant A were tested for mean difference using a two-tailed Mann–Whitney *U*-test with a significance criterion of *p* < 0.05.

Correlation coefficients and corresponding significance values between stimulation current intensity and VAS scores were calculated using Pearson correlation for all electrode pairs that had at least two supra-threshold VAS scores and at least one sub-threshold VAS score of 0 mm. Within each electrode pair, all sub-threshold VAS scores except that with the highest current intensity were ignored for statistical purposes.

## Results

### Electrode Localization

Peripheral mechanical stimulation evoked clear cortical responses in both participants (Figure 2A), allowing estimation of the functional locations of electrodes. The cortical responses (thumb sensation in Figure 2B; tongue sensation in Figure 2C) in participant A demonstrated a clear division between the more medial thumb-associated area and the more lateral tongue-associated area. Also, the types of somatosensations evoked by electrical stimulation to the different electrodes in participant A demonstrated a similar division between thumb-associated (blue arrows in Figure 3A) and tongue-associated (red arrows in Figure 3A) areas. PTs in participant A ranged from 1.9 to 2.2 mA (Figure 3A). PTs in participant B ranged from 1.6 to 2.1 mA, and 3 out of 8 electrode pairs stimulated did not evoke any reported sensation (Figure 3B).

Even under S1 stimulation, higher current intensities evoked movements which might be a result of current spread to the motor cortex. Evoked movements were typically muscle twitches or small movements. MTs in participant A ranged from 2.0 to 2.6 mA (Figure 3C). MTs in participant B ranged from 2.0 to 2.3 mA, and 5 of the 8 electrode pairs stimulated did not evoke any visible movement (Figure 3D).

In participant A, polarity-dependent differences in both PTs and MTs, of up to 0.3 and 0.4 mA, respectively, were observed within particular pairs of electrodes (Figures 3A,C). In participant A, PTs (mean ± SE 2.06 ± 7.19E-2 mA) were lower than MTs (2.43 ± 9.18E-2 mA) (two-tailed Mann–Whitney *U*-test, *p* = 0.0175, *U* = 6.00) (Figure 3E). Participant B’s PTs (1.94 ± 9.27E-2 mA) tended to be lower than her MTs (2.20 ± 0.100 mA), but the mean difference did not meet significance (two-tailed Mann–Whitney *U*-test, *p* = 0.143, *U* = 2.5) (Figure 3F).

### Linear Relationship Between Current Intensity and VAS Score

**FIGURE 4:**
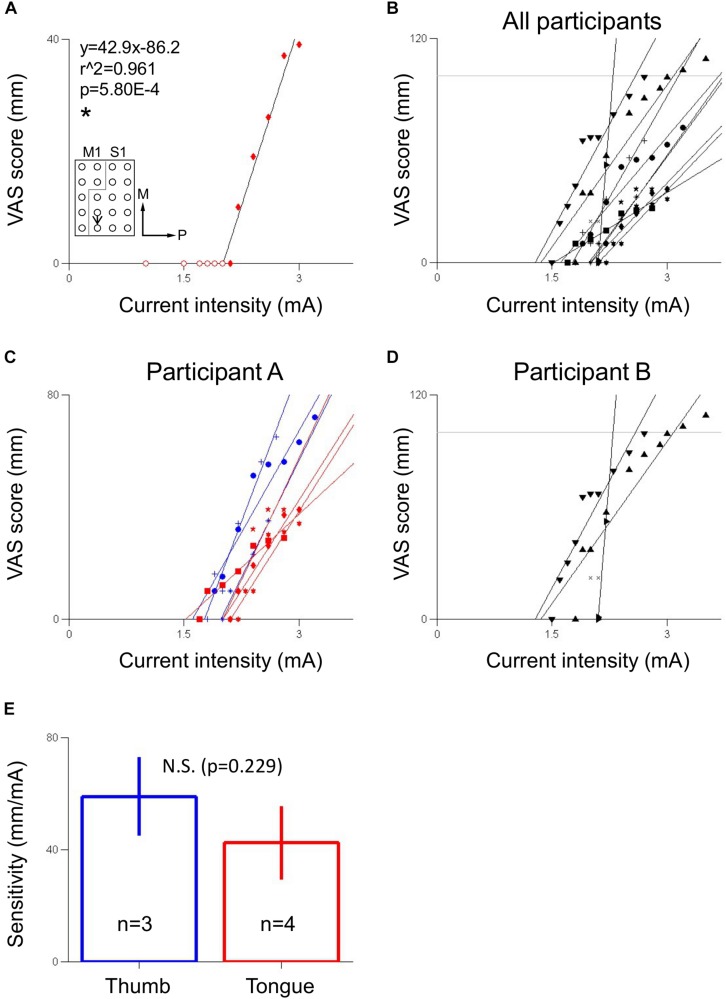
Relationship between S1 stimulation current intensity and VAS score of evoked sensation. **(A)** Electrode pair showing a linear relationship between visual analogue scale (VAS) score, which corresponded to sensation of a hard object on the tongue, and current intensity in participant A. Inset indicates location of stimulated electrode pair. Black arrow points from anode to cathode indicating stimulation polarity. Gray line indicates estimated location of central sulcus. Numerals in inset indicate coefficient of correlation (r^2^) and *p*-value (p). Equation describes VAS score (y) in mm as a function of current intensity (x) in mA. The slope of the line is the sensitivity of the electrode pair in mm/mA. ^∗^Indicates significance at the *p* < 0.05 level. Fitting ignores redundant-pre threshold points, indicated by hollow circles. Axes labels indicate medial (M) and posterior (P) directions. **(B)** VAS score versus current intensity for electrodes across both participants. **(C)** VAS score versus current intensity for electrodes in participant A, differentiated by perceived body area (blue: thumb, red: tongue). **(D)** VAS score versus current intensity for electrodes in participant B, corresponding to hand sensation. In **(B,D)**, horizontal gray line indicates the 100 mm “strongest sensation” endpoint of the VAS. In **(C,D)**, redundant pre-threshold points (open circles in **A**) have been removed for clarity. **(E)** Comparison of sensitivities between thumb and tongue areas in participant A using a two-tailed Mann–Whitney *U*-test with a significance criterion of *p* < 0.05. Bars and error bars indicates mean and standard error, respectively. N.S. indicates non-significance.

**FIGURE 5:**
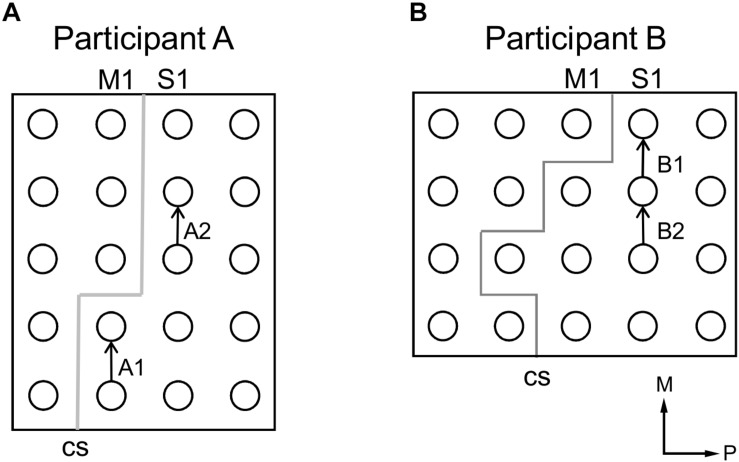
Electrode pairs selected for samples of participants’ verbal description of stimulation-evoked sensation (see Table 1). **(A)** Selected electrode pairs for participant A. **(B)** Selected electrode pairs for participant B. In **(A,B)**, arrows point from anode to cathode indicating stimulation polarity and gray lines indicate estimated locations of central sulci (CS). Axes labels indicate medial (M) and posterior (P) directions.

There was a clear, positive linear relationship between current intensity and VAS score in both participants within the range of current intensities tested (Figure 4). Out of all 11 electrode pairs analyzed, 10 were significantly correlated at the *p* < 0.05 level, while 1, which had three VAS-current intensity data points, did not. In all significantly correlated electrode pairs, r was greater than 0.9, and r^2^ was greater than 0.85, indicating a strong, positive linear relationship. In participant A, thumb sensation-related electrode pairs (mean ± SE 58.9 ± 7.10 mm/mA) tended to be more sensitive to current intensity than tongue sensation-related electrode pairs (42.4 ± 6.64 mm/mA) (Figure 4E), though this difference did not meet significance at the *p* < 0.05 level (two-tailed Mann–Whitney *U*-test, *p* = 0.229, *U* = 2.00). For electrode pair B1 (Figure 5), participant B exceeded the “strongest sensation” endpoint of the VAS, reporting a sensation corresponding to 109 mm at 3.5 mA (Figure 4D). Some electrode pairs, particularly those associated with tongue sensation, seemed to exhibit a saturation effect around the higher end of the range of the current intensities tested, showing a decreasing sensitivity as current intensity increased. However this phenomenon was not robust enough in the range of current intensities tested to warrant further analysis.

### Current Intensity-Dependent Differences in Evoked Sensation

Participant A described electrically-evoked sensations largely in terms of feelings of touch and numbness. He described qualitative features of the sensations that changed with current intensity, for both thumb-related and tongue-related sites. Participant B described electrically-evoked sensations largely in terms of pressure on the hand. Increases to current intensity generally caused her to describe increases in the intensity of the sensation; increased current would evoke sensation of stronger, wider, or faster pressure on the hand. It was demonstrated that current intensity has an impact on both intensity-related features such as and size and pressure, as well as more qualitative aspects corresponding to different types of somatosensation (Figure 5 and Table 1).

**TABLE 1:** Selected verbal descriptions of stimulation-evoked sensation given by participants.

**Participant**	**Electrode pair**	**Current intensity (mA)**	**Description of sensation**
A	A1	1.8^T^	*Sense of a flick on a narrow portion of the right side of the tip of my tongue. Feels like movement [on my tongue].*
A	A1	2.0	*The surface of the right side of the tip of my tongue feels numb. It’s as if I’m eating pineapple, but there’s no taste. The surface feels numb.*
A	A1	2.2	*The feeling [of numbness] on the right-side of the tip of my tongue spread from front to back.*
A	A1	2.8	*It feels like my tongue is swollen like after being bitten.*
A	A2	1.9^T^	*Not a feeling of being touched; a bad feeling [around the second joint of the thumb].*
A	A2	2.0	*Feels like a blood vessel pulsing [in my thumb], not like anything I’ve felt in the past. Feels like stimulation by a low frequency electrical stimulation therapy device around the second joint of the thumb.*
A	A2	2.5	*Outer side of my thumb feels anaesthetized.*
A	A2	2.7	*A slow dull touch on the opposite hand. Also, my finger feels swollen and thick.*
B	B1	1.9^T^	*The palm under my left little finger is being pressed. The spot moved a little inwards [to the center of my palm].*
B	B1	2.0	*It’s being pressed down in a few spots [on my palm] but the overall width is the same [as 1.9 mA].*
B	B1	2.5	*It feels faster. The width is the same.*
B	B1	3.0	*The width increased.*
B	B2	1.6^T^	*[no verbal description given by participant; VAS response only]*
B	B2	1.7	*I feel pressure at two spots on the [ulnar] side of my palm.*
B	B2	2.3	*It sped up, and got stronger [than at 1.9 mA]. The width is unclear.*
B	B2	2.7	*It got even faster, but the width is the same. It feels like something is really strongly pressing [into my skin].*

**For electrode pair definitions (see Figure 5). Original responses were given in Japanese. Text in square brackets was not said by participants but is included for clarity. ^*T*^Indicates PT for that electrode pair.**

## Discussion

These results demonstrate a quantitative analysis of sensation evoked by S1 electrical stimulation using ECoG arrays in humans. A linear relationship between stimulation current intensity and intensity of sensation was observed in both for hand and tongue representation of S1; increasing supra-threshold stimulation current intensity resulted in a proportional increase in intensity of sensation. This opens the potential for clinical application in developing somatosensory neuroprosthetics for clinically plausible BMI.

VAS response curves enable a quantitative determination of what intensity of sensation should be expected for a given stimulation current intensity before stimulation is applied, allowing for easier calibration of stimulation parameters in design of somatosensory neuroprosthetics. The sensitivity of VAS response to current intensity could inform what current intensity to use in different somatotopic regions of S1 to evoke sensation of a particular desired intensity; the non-significant tendency in participant A of thumb-related electrode pairs to be more sensitive than tongue-related electrode pairs suggests that different somatotopic regions of S1 may have different sensitivities to electrical stimulation (Figure 4), although a larger sample size and testing of more somatotopic regions of S1 are needed to elucidate this phenomenon more fully. Although ethical and safety considerations, including avoiding inducing epilepsy by monitoring for after-discharges, necessitated the current intensity be generally increased from low to high, it is possible that randomizing the order of the current intensities to be tested may provide more robust results. Furthermore, especially for the evaluation of sensory threshold, more detailed psychophysical methods, such as two-alternative forced-choice or yes/no task (Flesher et al., 2016; Devecioglu and Guclu, 2017), might be used to derive psychometric functions and to minimize or control cognitive bias. However, because such psychophysical methods necessitate a larger number of stimulation trials and a longer experiment time, such methods may be difficult to implement, both practically and ethically, in the clinical context of epilepsy patients with implanted electrodes for preoperative evaluation. Additionally, VAS was suitable here because this experiment focused on the quantitative relationship between the intensity of electrical stimulation and the perceived intensity and quality of the evoked sensation. Perhaps in future experiments a range of non-epileptogenic current intensities could be preliminarily ascertained by gradual increase of the current intensity as was done here, and then current intensities could be randomly selected from within that range for another round of stimulation, the results of which could then be used to construct the VAS response curve and/or another psychophysical method.

A study on S1 stimulation via ECoG in humans (Johnson et al., 2013) found that changing either stimulation frequency or current intensity changed only the intensity of the sensation, without significant change in the quality of the sensation. The results in this study seem to partially conflict with those findings; participant B indeed described mainly changes to intensity, but participant A described clear differences in quality of sensation as current intensity was varied (Figure 5 and Table 1). These results are commonly observed in some recent studies using intracortical microelectrode and surface microelectrode (Flesher et al., 2016; Hiremath et al., 2017). More rigorous investigation of the relationship between stimulation parameters, electrode characteristics, and stimulation sites, neural recruitment by stimulation, and quality of evoked sensation is needed to determine the factors that contribute to the presence or absence of these qualitative changes at a given stimulation site.

Ethical and clinical considerations limited the range of current intensities that could be used for S1 stimulation. Despite the overall linearity of VAS response to current intensity, there was some indication toward the upper limit of the ranges of current intensity tested that as current intensity increased, there was a decrease in slope of the VAS response curve, especially in participant A’s tongue-related sites (Figure 4C) and participant B (Figure 4D). This is suggestive of a saturation effect; as current intensity increases and a wider area of the S1 containing the somatotopy of the associated body area is activated (Haglund et al., 1993), the number of somatotopically relevant neurons still available for recruitment may decrease as the percentage of recruited neurons tends toward 100%. The resultant decrease in the number of additional neurons recruited by every increase in stimulation current intensity, then, may lead to a decrease in the perceived difference in intensity of subsequent evoked sensations. This could explain why, in participant A, this saturation phenomenon was observed more in tongue-related electrode pairs than in thumb-related ones: the tongue-related area in the human S1 is physically smaller than the thumb-related area (Nakamura et al., 1998), and thus would approach 100% recruitment at a lower current intensity.

In addition to evoking sensation of particular body areas, S1 electrical stimulation also drove small movements of the same general body areas, in accordance with previous findings (Penfield and Boldrey, 1937). In participant A the threshold current intensity for evoked movement was higher than that for evoked sensation (Figure 3E). In participant B this relationship appeared to exist as in participant A, but, likely owing to the relatively small number of electrodes pairs that evoked either sensation or movement, this relationship was not found to be statistically significant (Figure 3F). Assessment of this phenomenon could prove critical in the calibration of S1 stimulation parameters for neuroprosthetics or psychophysical experiments; to produce a target level of sensation per unit of input sensory signal, the mathematical transformation of that sensory signal into current intensity would require knowledge of the sensitivity of the VAS response curve, including any non-linearity such as saturation effects. The potential for a motor response puts a limit on the current intensity that can be applied when the only desired effect of stimulation is evoked sensation. This, along with the saturation considerations detailed above, could be crucial factors to consider in designing future S1 stimulation experiments or neuroprosthetics for clinical use.

It should be noted that some of the stimulation below the motor threshold might have evoked some very weak muscle contractions which that were not noticed by the experimenter. Although the possibility that the evoked sensations originated from these weak muscle contractions cannot be ruled out completely, the verbal descriptions of the evoked sensations seem consistent with pressure or cutaneous mechanoreceptor-type sensations, which are unlikely to be stimulated by such weak muscle contractions. Furthermore, the established physiological role of S1 suggests that the evoked sensations at least primarily originated from the electrical stimulation to S1.

Notably, some electrode pairs did not induce artificial sensation. Electrical conditions may differ between electrode pairs. For example, some electrodes might not contact the cortical well as well as others. This larger distance between the electrode and the cortical surface might lead to more current spread over the cortex via the cerebrospinal fluid. Moreover, as seen in the case of stimulation using intracortical electrodes, stimulation in deeper layer tends to decrease detection thresholds (DeYoe et al., 2005; Tehovnik and Slocum, 2009; Koivuniemi and Otto, 2012). The distance between the cortex and the electrode might also affect the effective depth of the electrical stimulation. For these electrode pairs, artificial sensation might have been potentially evoked using higher current intensity. However, stimulation current intensity was limited by the clinical factors such as the need to avoid epileptic discharges as a result of stimulation. Although in practice it is difficult to control surface electrode location precisely, these results demonstrated that electrical stimulation via surface electrodes induced artificial sensation with properties similar to those of artificial sensation induced by electrical stimulation via intracortical microelectrodes.

In participant A, S1 stimulation revealed a clear division between more medial thumb-related areas and more lateral tongue-related areas (Figures 3A,C), corresponding to the widely known somatosensory homunculus (Penfield and Boldrey, 1937). This general layout was also observed in the spatial distribution of cortical responses evoked by mechanical stimulation (Figures 2B,C). However it is clear that the tongue-related areas as determined by cortical responses to mechanical stimulation were more medial than those determined by S1 electrical stimulation. There is a reasonable explanation for this discrepancy. At some cortical sites, for example the more posterior electrodes in participant A, the cortical response to mechanical stimulation appeared to be a decrease in high-gamma power (Figure 2D), rather than the increase observed elsewhere. Further, stimulation via ECoG electrodes recruits a large number of neurons en masse, whereas external mechanical stimulation produces more nuanced recruitment patterns, which may include the aforementioned suppression effects, which correspond to physiological somatosensation. Thus, it is difficult to compare, from both mathematical and physiological perspectives, the recording of physiological activity at single electrodes versus data based on bipolar stimulation of electrode pairs. This difference can also explain the somewhat unusual sensations evoked by S1 stimulation (Table 1). The polarity-dependent differences in both PT and MT in individual electrode pairs observed in subject A (Figures 3A,C) suggest that stimulation polarity has an effect on neural recruitment, presumably based on the cytoarchitecture of the cortex.

It is difficult to induce artificial somatosensation using non-invasive percutaneous cortical stimulation such as transcranical magnetic or electrical stimulation. Invasive cortical stimulation using penetrating electrodes has been demonstrated to induce artificial sensation in a spinal cord injury patient (Flesher et al., 2016). Electrical stimulations through the intracortical micro-electrode arrays requires a lower current intensity less than 100 μA to evoke somatosensory sensations (Flesher et al., 2016), while cortical surface stimulations required mA order to induce somatosensory sensation (Figure 3). The intracortical micro-electrodes were also useful to evoke precise and discrete somatosensory sensations (Flesher et al., 2016) and to decode motor information, but the stability of the electrodes makes their implementation in a clinical context difficult. The stimulation area is also limited with intracortical micro-electrodes while ECoG allows coverage of a larger cortical area beyond different body representations. ECoG therefore appears to be a well-balanced technique that allows greater cortical coverage while remaining less invasive than cortex-penetrating needle electrodes, and as such is useful in medical contexts where minimal invasiveness is desired (Yanagisawa et al., 2012; Matsushita et al., 2018). Therefore, ECoG offers one of the most clinically feasible options, being less invasive, having superior long-term stability, and being less technically difficult to implement (Rubehn et al., 2009; Chao et al., 2010; Shin et al., 2012; Nakanishi et al., 2017) compared with other invasive stimulation methods. These advantages should facilitate the development of the clinically plausible somatosensory BMI.

## Ethics Statement

This experimental protocol was approved by the Ethics Committees of the Osaka University Hospital (Approval No. 14353), the National Institutes for Physiological Sciences (Approval No. 16B004), and the Tokyo Metropolitan Institute of Medical Science (Approval No. 17-2) and carried out in accordance with the Declaration of Helsinki. Participants or their guardians provided written, informed consent to the experimental procedures and to the use of their data for academic study.

## Author Contributions

YN and TY conceived and designed the experiments. TY, YN, SO, KE, MT, and HK performed the experiments. SK, YN, and TY analyzed the data and wrote the manuscript. SK and YN prepared the figures.

## Conflict of Interest

The authors declare that the research was conducted in the absence of any commercial or financial relationships that could be construed as a potential conflict of interest.
